# Influence of recovery strategies upon performance and perceptions following fatiguing exercise: a randomized controlled trial

**DOI:** 10.1186/s13102-017-0087-8

**Published:** 2017-12-28

**Authors:** Fiona Crowther, Rebecca Sealey, Melissa Crowe, Andrew Edwards, Shona Halson

**Affiliations:** 1 College of Healthcare Sciences, James Cook University, Townsville, QLD Australia; 2 College of Healthcare Sciences, James Cook University, Townsville, QLD Australia; 3 Division of Tropical Health and Medicine, James Cook University, Townsville, QLD Australia; 4 Sport & Health Sciences, University of St Mark and St John, Plymouth, Devon UK; 5 Australian Institute of Sport, Canberra, ACT Australia

**Keywords:** Recovery, Sports science, Performance

## Abstract

**Background:**

Despite debate regarding their effectiveness, many different post-exercise recovery strategies are used by athletes. This study compared five post-exercise recovery strategies (cold water immersion, contrast water immersion, active recovery, a combined cold water immersion and active recovery and a control condition) to determine which is most effective for subsequent short-term performance and perceived recovery.

**Methods:**

Thirty-four recreationally active males undertook a simulated team-game fatiguing circuit followed by the above recovery strategies (randomized, 1 per week). Prior to the fatiguing exercise, and at 1, 24 and 48 h post-exercise, perceptual, flexibility and performance measures were assessed.

**Results:**

Contrast water immersion significantly enhanced perceptual recovery 1 h after fatiguing exercise in comparison to active and control recovery strategies. Cold water immersion and the combined recovery produced detrimental jump power performance at 1 h compared to the control and active recovery strategies. No recovery strategy was different to the control at 24 and 48 h for either perceptual or performance variables.

**Conclusion:**

For short term perceptual recovery, contrast water therapy should be implemented and for short-term countermovement power performance an active or control recovery is desirable. At 24 and 48 h, no superior recovery strategy was detected.

**Trial registration:**

Retrospectively registered; ISRCTN14415088; 5/11/2017.

## Background

High performance athletes employ a variety of strategies [1, 2] with the intention of accelerating their recovery [3]. Non-elite levels of athlete have also been shown to undertake a number of different recovery strategies post-exercise [4], potentially to decrease soreness and improve subsequent performance. The efficacy of numerous recovery strategies has been explored in scientific studies and also in practical sport applications, with some strategies being used without compelling supportive evidence [3, 5, 6].

Water immersion recovery strategies such as cold water immersion (CWI) and contrast water therapy (CWT) are used by athletes across a range of competition levels [4] to enhance post-exercise recovery [6–8]. Cold water immersion reportedly minimises muscle oedema and provides analgesic effects post-exercise [8]. Contrast water therapy is the alternation between hot and cold water [6] and is reported to decrease lactate accumulation [9], inflammation, oedema, pain and muscle stiffness [6]. The common explanation for CWT effectiveness is the pumping action of circulating blood, which is caused by alternation between vasodilation and vasoconstriction in response to hot and cold water [6].

A number of reviews are inconclusive as to whether CWI or CWT are effective recovery strategies following exercise and sport [3, 5, 6]. Other recent reviews have shown CWI to reduce delayed onset muscle soreness [10] and fatigue [11]. Alternatively, Bieuzen and colleagues [2] found CWT to be no better than CWI, warm water immersion, active recovery (ACT) and stretching, although better than passive rest. In addition, Torres and colleagues [12] found CWI, ACT and stretching to be generally not effective or inconsistent in improving muscle soreness or strength.

An ACT recovery is a simple and commonly used technique that involves the completion of low intensity exercise following prior exercise, and has been suggested to increase blood flow and range of motion which may lead to the acceleration in the decrease in interstitial creatine kinase [13]. Active recovery may also allow reoxygenation of blood thorough increased alveolar gas exchange as a consequence of elevated metabolism compared to passive recovery strategies. Based on the reported effectiveness of CWI and ACT, these two strategies were combined and investigated.

A limited number of studies have investigated the combination of CWI and ACT recovery strategies (COMB), with mixed results [14–16]. This combined recovery has been shown to be effective at removing blood lactate [15, 17], and eliciting positive perceptions of recovery [17, 18].

The purpose of this unique study is to investigate the effects of five recovery strategies (CWI, CWT, ACT, control (CONT) and COMB) on indicators of performance (repeated sprint ability and repeated countermovement jump) sit and reach flexibility, and perceptual recovery (Daily Analysis of Life Demands of Athletes (DALDA) questionnaire, muscle soreness scale and Total Quality Recovery (TQR) scale) following fatiguing exercise in non-elite athletes. The effects of these five recovery strategies upon performance and perceptual recovery to the authors’ knowledge have not been investigated. The findings from this investigation may provide non-elite team sport athletes and coaches with up-to-date information to assist with informed decision making about their recovery choices. Water immersion strategies were hypothesised to be superior to ACT and CONT for performance and perceptual indices of recovery over the 48 h time period.

## Methods

Thirty-four recreationally active, uninjured, apparently healthy males voluntarily participated in the study (mean ± SD; age: 27 ± 6 years; height: 180 ± 8 cm; weight: 80 ± 9 kg; VO_2_max: 43 ± 6 ml/kg/min). All participants were able to complete the fatiguing exercise, participated in regular aerobic exercise and were not elite athletes. Five participants were unable to complete all scheduled testing sessions, due to external factors unrelated to testing; however their data were included for completeness in quantitative analysis of the completed recovery protocols. Contact sport athletes were excluded from the study due to the potential for muscle damage caused by external sports participation. Participants were instructed to abstain from exercise and alcohol 24 h before the first session until the conclusion of the 48 h post testing session, and to abstain from food 2 h and caffeine 4 h prior to sessions. Exercise diaries were completed throughout the testing period and were analysed to confirm consistency of exercise throughout the testing period and adherence to the research project instructions. Participants were informed of the procedures to be undertaken and provided written informed consent prior to participation. Ethics approval was granted by the Human Ethics Committee of James Cook University (H5415).

Participants performed two familiarisation sessions. The first session included a standardised, generalised warm up (jog and dynamic exercises), 3 × 20 m maximal sprint runs on a grassed area for the determination of peak speed, and a practice of the repeated sprint ability [19, 20] and countermovement jump tests adapted from Elias and colleagues [19] and King and Duffield [21]. The repeated sprint ability test has a reported coefficient of variation of 2.3%, and total sprint time has a strong correlation to fastest 20 m sprint time (*r* = 0.66) in research conducted using well-trained AFL players on a wooden sprung floor [22]. During the second session participants completed the generalised warm up followed by a practice of the sit and reach flexibility test [23], completion of the multi-stage fitness test to assess aerobic capacity [24], practice of the fatiguing exercise and familiarisation with the DALDA scale [25], muscle soreness scale [26] and TQR scale [27]. The five recovery protocols were also explained at this time.

**Fig. 1:**
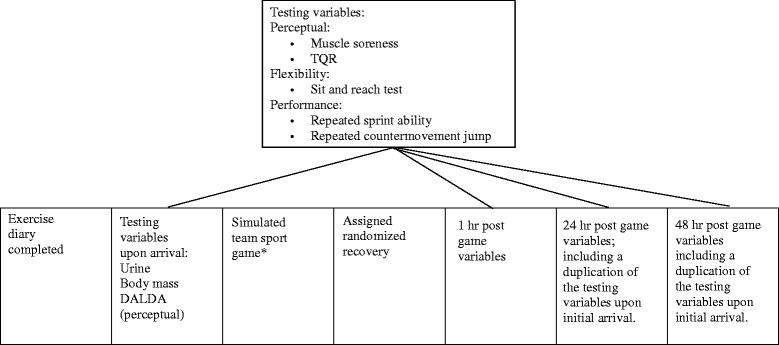
Timing of variable assessment during testing days. *HR recorded throughout and RPE at the conclusion

See Fig. 1 for a schematic diagram of the timing and variable testing throughout the testing days. At the start of each testing session, participants were assessed for hydration status via urine specific gravity measurement with the use of a handheld refractometer (John Morris Scientific Pty Limited), had their body mass measured for subsequent power calculations for the countermovement jump test, and completed the DALDA questionnaire, muscle soreness scale and TQR scale. The DALDA questionnaire lists a series of life-stress and symptoms of stress, where participants label each item with a letter; “a” means worse than normal, “b” means normal and “c” indicates better than normal [25]. The muscle soreness scale was a 10-point Likert scale from 0 (no soreness) to 10 (very very sore) [26]. The TQR was a scale that ranged from 6 (below very very poor recovery) to 20 (above very very good recovery) [27]. For these two perceptual scales, participants were allowed to give numbers that were not whole numbers. A generalised warm up (jog and dynamic exercises) was undertaken prior to completion of the sit and reach test, repeated sprint ability test and the counter-movement jump test. The sit and reach test was performed 3 times, with the best measurement recorded for analysis. Participants undertook the repeated sprint ability test, which included a maximal 20 m sprint every 30 s with six repetitions [19, 20]. The countermovement jump test included five jumps of maximal height on a mat, one jump every 15 s [19, 21], with jump height and power recorded. Sprint and jump performance were measured with the Swift timing gates and mat (Swift Performance Equipment, QLD, Australia).

**Fig. 2:**
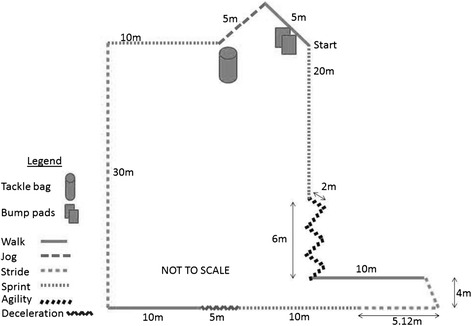
Diagram of the simulated team-game fatiguing circuit adapted from Singh and colleagues [27] and Bishop and colleagues [28]

Participants then completed a 3 × 15 min simulated team-game circuit adapted from Singh and colleagues, [28] and Bishop and colleagues, [29] as the fatiguing exercise protocol. The fatiguing exercise involved a circuit undertaken each min which included sprinting, striding, jogging, walking and agility, with bag tackles completed on every fifth rotation [28] and bumps (participants were contacted with bump pads three times on each side of the body as adapted from [28] on the 15th rotation (Fig. 2). After 15 rotations participants rested for five min before repeating the process two more times. Heart rate was monitored throughout (Polar heart rate monitor, Polar Electro Oy, Kempele, Finland) and Borg’s RPE [30] was recorded at the completion of the third round.

Following a ten min rest, participants completed a five min jog at 20% peak speed (adapted from [31]), with peak speed calculated from the maximal sprints in the first familiarisation session. This jog was implemented for practical reasons, as many teams would undertake an active component prior to recovery. Participants then undertook their randomly assigned recovery protocol, CWI, CWT, ACT, COMB or CONT. All recovery protocols were undertaken for 14 min. Cold water immersion included being seated in an inflatable bath (iCool Sport, Shenzhen, China), with shoulders immersed at a temperature of 15 °C [31]. Contrast water immersion included alternating between a cold bath set to 15 °C and a hot bath set to 38 °C (iCool sport, Shenzhen, China), both to shoulder immersion depth [31], with participants instructed to change baths every 1 min. Active recovery included outdoor jogging around a marked and measured grass track at 35% peak speed as adapted from King and Duffield [21] with continual feedback to maintain the desired speed. The COMB recovery was performed as per the cold water immersion protocol with the addition of low intensity cyclic leg movement inside the cold bath. Running was not able to be incorporated into the recovery due to inadequate facilities. Participants’ heart rate was recorded every 10 s and averaged 48 ± 5% of their maximum heart rate. The CONT protocol involved participants passively sitting on a chair, with as little movement as possible. All recovery protocols and testing procedures were performed outdoors at natural environmental temperatures with no significant difference found over the five sessions for temperature (*p* = 0.230) (average range over five conditions 22.6 °C - 23.9 °C) and humidity (*p* = 0.955) (average range over five conditions 71.9% - 73.9%).

After each recovery protocol, participants undertook seated rest until 1 h elapsed from completion of the fatiguing exercise. Participants then completed the TQR and muscle soreness scale, performed the standardised warm up and completed the sit and reach, repeated sprint ability and countermovement jump tests (approximately 30 min combined). Participants drank water ad libitum throughout the testing day. No food was provided during the 3 h session to allow for better test controllability. The entire duration of the testing session was approximately 3 h.

Participants returned at 24 and 48 h post completion of the fatiguing exercise for the following tests: urine specific gravity, DALDA, muscle soreness scale, TQR; and the same generalised warm up, sit and reach, repeated sprint ability and counter-movement jump tests. At the conclusion of all testing participants were asked which recovery strategy they thought was most effective and which was least effective and to give reasons why. Participants were blinded to the results of the performance tests. The entire testing process was repeated each week until participants had completed all five randomly ordered recovery strategies (excluding those who were unable to finish). Participants performed testing at approximately the same time each day.

### Statistical analyses

Data were analysed using the Statistical Package for Social Sciences (IBM SPSS Incorporation, Version 22, Chicago, Ill, USA) via two-way (time x recovery) repeated measures ANOVA and post hoc Tukey HSD tests. Repeated sprint ability and relative average and best power at 1 h post were assessed between weeks for order of effect. Data were presented as means ± standard deviation (SD) with alpha set at 0.05. All results are interaction results unless specified. The following variables were analysed; RPE, average HR, hydration, DALDA scale, muscle soreness, TQR, best sit and reach performance, total repeated sprint time, relative (normalised for mass) average and peak jump power performances. The following recovery-related components of the DALDA scale were analysed; muscle pains; need for rest; recovery time; unexplained aches; between session recovery; and swelling. Incomplete data points were accounted for by using the recovery and time specific average.

## Results

Order of effect testing indicated no difference for 1 h post repeated sprint ability between the weeks (*p* = 0.243; week 1 21.8 ± 2.5 s, week 2 21.9 ± 1.3 s, week 3 21.6 ± 1.2 s, week 4 22.2 ± 1.5 s and week 5 22.3 ± 1.5 s). For power measures no differences were found across weeks (relative average *p* = 0.573 and best *p* = 0.606 respectively, week 1 15.9 ± 2.2 W/kg and 16.4 ± 2.1 W/kg, week 2 15.8 ± 2.2 W/kg and 16.3 ± 2.2 W/kg, week 3 15.7 ± 2.3 W/kg and 16.2 ± 2.2 W/kg, week 4 15.6 ± 2.2 W/kg and 16.2 ± 2.2 W/kg and week 5 15.6 ± 2.3 W/kg and 16.1 ± 2.3 W/kg), confirming there is no order of effect or adaptation.

The mean between-trial coefficient of variation for countermovement jump test (average and peak), sit and reach, TQR, RPE and average HR across all five sessions was 6%, 10%, 4%, 8% and 4%, respectively, showing high reproducibility and comparability of the test conditions, load and participant effort. Further, average RPE values post each fatiguing exercise by condition were as follows CWI 16 ± 1.6; CWT 16 ± 2.3; ACT 16 ± 2.0; COMB 16 ± 1.8 and CONT 16 ± 2.2. Average HR values during fatiguing exercise were as follows CWI 168 ± 11.3 bpm; CWT 164 ± 10.9 bpm; ACT 165 ± 10.3 bpm; COMB 165 ± 8.4 bpm and CONT 165 ± 11.6 bpm. The mean between-trial coefficient of variation for hydration across all sessions was 0.8% (average range over five conditions 1.02–1.03), therefore it is unlikely to impact upon results.

One hour following fatiguing exercise, CWT elicited superior perceptions of recovery (muscle soreness 2.5; TQR 15.7) in comparison to ACT (muscle soreness 3.8; TQR 13.7) and CONT (TQR only 14.2). At 1 h CWI (CMJ relative peak power 15.9; relative average 15.4) and COMB (CMJ relative peak power 15.9; relative average 15.4) recovery strategies showed detrimental performance results in comparison to ACT (CMJ relative peak power 16.7; relative average 16.1) and CONT (CMJ relative peak power 16.5; relative average 16.0). However, there was no difference between the five recovery strategies at 24 and 48 h for perceptual and performance recovery.

As an overall time effect, the response to the DALDA scale muscle pain was significantly worse at 24 h post-exercise in comparison to 48 h (*p* < 0.001). Swelling was significantly greater at 48 h (*p* = 0.038) compared to 24 h via the DALDA perceptual scale. A main effect of recovery mode was also evident for DALDA item “need for rest” with CWI eliciting less need for rest than CWT (*p* = 0.022).

A significant main effect for time occurred for muscle soreness with scores significantly higher at 1 h and 24 h compared to baseline and 48 h (Table 1). Muscle soreness in the CWI and ACT recovery strategies showed no difference to the CONT with significantly higher muscle soreness scores than baseline at 1 h and 24 h (Table 1). At 48 h, ACT showed no difference to CONT with both recovery strategies showing values significantly better than their respective 1 h readings. At 1 h, CWT resulted in less muscle soreness than ACT (Table 1). There was no difference in muscle soreness across time for the CWT and COMB protocols.

**Table 1:** Perceptual and performance measures assessed at baseline and 1 h, 24 h and 48 h after fatiguing exercise for each of the different recovery strategies

Measures Recovery strategy					
	Control (CONT)	Cold (CWI)	Contrast (CWT)	Active (ACT)	Combined (COMB)
Muscle soreness					
Baseline	1.7 ± 1.8	1.8 ± 2.0	2.1 ± 1.9	1.5 ± 1.6	2.0 ± 2. 1
1 h post^e^	3.6 ± 2.2^a^	3.3 ± 2.0^b^	2.5 ± 1.7	3.8 ± 1.7^ac^	3.0 ± 1.8
24 h post^e^	3.2 ± 1.9^b^	3.3 ± 2.1^b^	2.9 ± 1.8	3.3 ± 1.8^b^	2.7 ± 1.5
48 h post	2.0 ± 1.7	2.4 ± 1.7	2.5 ± 1.6	2.4 ± 1.9	2.1 ± 1.7
TQR					
Baseline	16.3 ± 2.0	16.5 ± 2.3	16.3 ± 2.5	16.5 ± 2.3	16.2 ± 2.3
1 h post^e^	14.2 ± 2.5^ac^	14.4 ± 2.5^a^	15.7 ± 1.9	13.7 ± 2.5^ac^	15.0 ± 2.1
24 h post^e^	14.3 ± 2.6^a^	15.6 ± 2.3	15.2 ± 1.8	15.0 ± 2.7^b^	15.6 ± 2.0
48 h post	15.9 ± 2.3	16.0 ± 2.1	15.9 ± 1.7	15.7 ± 2.5	16.1 ± 1.8
Sit and Reach (cm)					
Baseline	31.7 ± 8.1	32.1 ± 9.0	31.8 ± 9.3	31.8 ± 9.0	32.0 ± 9.7
1 h post	32.2 ± 7.8	31.8 ± 9.2	32.3 ± 9.1	32.1 ± 8.5	32.2 ± 8.9
24 h post	31.4 ± 8.6	31.3 ± 9.5	31.9 ± 9.7	31.9 ± 9.3	31.8 ± 9.7
48 h post	32.5 ± 8.5	31.5 ± 9.2	31.7 ± 9.5	31.9 ± 9.1	31.7 ± 9.8
Total sprint time (s)					
Baseline	21.4 ± 1.7	21.0 ± 1.0	21.3 ± 1.1	21.2 ± 1.2	21.4 ± 1.3
1 h post^ef^	21.9 ± 2.4	22.0 ± 1.3	21.8 ± 1.4	21.6 ± 1.4	22.3 ± 1.5
24 h post	21.4 ± 1.4	21.5 ± 1.3	21.5 ± 1.4	21.4 ± 1.3	21.4 ± 1.1
48 h post	21.6 ± 1.8	21.2 ± 1.4	21.4 ± 1.3	21.2 ± 1.3	21.2 ± 1.0
CMJ relative peak power (W/kg)					
Baseline	16.5 ± 2.1	16.6 ± 2.2	16.4 ± 2.1	16.8 ± 2.3	16.4 ± 2.1
1 h post^g^	16.5 ± 2.3	15.9 ± 2.1^ad^	16.2 ± 2.0	16.7 ± 2.4	15.9 ± 2.1^d^
24 h post	16.5 ± 2.2	16.3 ± 2.1	16.2 ± 2.3	16.5 ± 2.4	16.2 ± 2.2
48 h post	16.4 ± 2.3	16.4 ± 2.4	16.2 ± 2.1	16.6 ± 2.5	16.4 ± 2.3

Interaction effects*:* ^a^Significant difference in comparison to respective baseline and 48 h post fatiguing exercise values. ^b^Significant difference from respective baseline measures. ^c^Significant difference in comparison to contrast recovery. ^d^Significant difference in comparison to active and control recovery strategies. Main time effects: ^e^Significant difference in comparison to baseline and 48 h post fatiguing exercise values. ^f^Significant difference in comparison to 24 h post fatiguing exercise values. ^g^Significant difference from baseline

The TQR demonstrated a significant main effect for time with recovery rates significantly lower at 1 h and 24 h compared to baseline and 48 h, with TQR ratings restored to baseline levels by 48 h (Table 1). The ACT and CWI TQR ratings at 1 h were no different from CONT with significantly decreased ratings from their respective baseline and 48 h values (Table 1). At 24 h ACT and CONT were both still reduced in comparison to baseline and 48 h (CONT only). ACT and CONT were also found to have significantly reduced TQR at 1 h in comparison to CWT. In contrast, the COMB and CWT protocols did not show significant decreases in TQR as a result of the fatiguing exercise across any time points.

No change in sit and reach performance was found across recovery strategies or times (Table 1). Total sprint times were significantly slower at 1 h in comparison to baseline, 24 h and 48 h (main effect for time) with no interaction or recovery effects evident (Table 1).

**Fig. 3:**
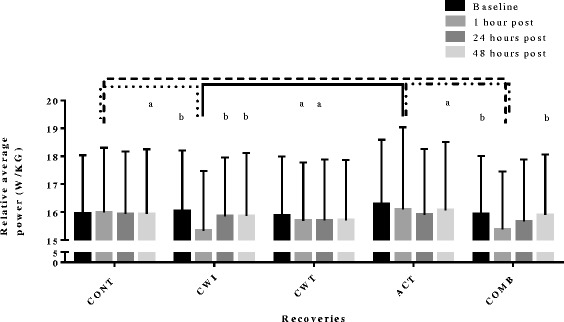
A comparison of countermovement jump relative average (±SD) power of all recovery strategies across all time points. **a** Significant difference between. **b** Significantly different from respective 1 h post values

A main effect for recovery was found for average and peak power, with ACT found to significantly improve jump performance variables in comparison to COMB and CWT (peak only). A main effect for time occurred for jump power with a significant reduction at 1 h compared to baseline (Table 1). Jump power variables at 1 h were significantly reduced compared to other time points for CWI (compared to baseline, 24 h (excluding peak power) and 48 h), and average power after COMB (compared to baseline and 48 h) with no effect of time evident for CWT, ACT or CONT (Table 1 and Fig. 3). CWI and COMB resulted in significantly reduced power (average and peak) at 1 h compared to CONT and ACT (Fig. 3).

At the conclusion of all protocols, CWT was rated as the most effective recovery strategy by the most participants (50%), followed by COMB and CWI (29% each). The top response given for why these recovery strategies were favoured was “felt better/good”, with “decrease in muscle soreness” also noted for CWI. Participants rated CWI the least effective recovery strategy (30%) for reasons such as “felt bad for the day after”, followed by ACT (26%), with the most common responses of “felt like more exercise” and “felt stiff”.

## Discussion

For the first time to the authors’ knowledge, this study compared a variety of post-exercise recovery protocols with differing effects on perceptions of recovery and subsequent performance measured over a 48 h period, to determine if there is a superior recovery strategy for non-elite athletes. The hypothesis that water immersion strategies would be superior to ACT and CONT for performance and perceptual recovery over the 48 h was only partially supported with CWT eliciting superior perceptions of recovery 1 h post exercise in comparison to ACT and CONT.

Athletes indicated that the CWT was the most positively perceived perceptual recovery strategy. This is most likely because CWT produced significantly reduced perceptions of muscle soreness and TQR ratings at 1 h in comparison to CONT (TQR only) and ACT. Similar findings have been reported with CWT producing better perceptual recovery following anaerobic exercise in comparison to ACT and CONT recovery strategies for state-level athletes [32], and CWT producing superior perceptual benefits of recovery in elite netball athletes following a fatiguing netball circuit in comparison to CONT [33]. Another previous study reported reduced perceptions of recovery 48 h post CWT in comparison to COLD [20], although this study utilised elite athletes. Despite the positive TQR and muscle soreness results in the current study, after CWT participants noted a significantly higher “need for rest” (DALDA) in comparison to CWI. Reasons for this response are not immediately clear. The current study found an improved perceptual rating after CWT which could be due to the vasodilation and vasoconstriction of capillaries which may cause a pumping effect, shunting metabolites out of the muscles and carrying new proteins and enzymes to the muscles [34], which may have assisted with perceived recovery. It is believed from participant feedback that the reason CWT was favoured over all other recovery strategies was due to the inclusion of heat. Participants often noted CWT having a relaxing, therapeutic effect upon the body, which has been supported by other authors [35]. The improved perceptual recovery after CWT may also be partially a “placebo” effect due to the large use of CWT within society and its assumed effectiveness. The therapeutic immediate post-treatment effects of CWT may be the primary factor in the common perception of the effectiveness of this strategy among athletic groups.

In the present study participants rated COMB as the second most effective recovery strategy. A number of previous studies of different populations of athlete support the positive perceptual findings of a combined ACT and CWI recovery [17, 18]. Mechanisms suggested as to why a COMB protocol is more effective in preventing decreased perceptual recovery than CWI, ACT and CONT include the action of hydrostatic pressure influencing an oscillating shift in blood volume due to movement of the lower limb [17], which may assist with increasing blood flow and thus greater perceptual benefits. Studies have found a combined recovery of ACT and CWI to remove lactate faster than CONT [15, 17] in similar population athletes. Furthermore, a COMB recovery may cause a reduction in neuron transmission speed within the body, resulting in decreased experienced pain [36]. This mechanism might explain the common analgesic effects reported for cold water immersion strategies [37], and may alleviate some sensations associated with tiredness. Christie and colleagues [38] found that volunteers cycling in water in comparison to the same cycling protocol on land increased central blood volume and decreased vascular resistance. Furthermore, a COMB strategy may assist to reduce muscle soreness and sensations of fatigue caused by oedema, faster than a land based active recovery [17]. Rapid post-exercise cooling strategies such as COMB, may provide a means to restore homeostasis and reduce intramuscular temperature [39]. The use of COMB after finishing exercise may therefore result in a better perception of recovery than other recovery strategies, due to increased hydrostatic pressure and analgesic effects.

In the current investigation CWI caused participants to have significantly less need for rest. In contrast, other perceptual measures were shown to be decreased after CWI in comparison to rest, with participants still noting significantly worse perceptual recordings at 24 h in comparison to baseline. In contrast Ingram and colleagues [40] found CWI to positively influence perceptions of muscle soreness at 24 h in comparison to CONT and CWT in similar athletes to those utilised in this study. Bailey and colleagues [41] also found CWI to significantly reduce muscle soreness ratings at 1, 24 and 48 h in similar athletes to those utilised in this study. Cold water immersion also produced significant perceptual benefits in a number of other studies that utilised high performance athletes [20, 42]. As previously stated CWI treatments are considered to be effective for perceptual recovery due to enhanced lactate removal, hydrostatic pressure and analgesic effects. In the present study, approximately 1 in 3 participants indicated CWI was the least effective, with some participants reporting feeling numb, stiff and sore. This was reported not only immediately after immersion, with one participant specifically stating soreness 1 day post testing. Another participant noted unusual muscle cramps between their 24 and 48 h follow up sessions. These statements from participants’ support why they did not perceive benefit from the CWI protocol. The differences in recovery protocols may have led to the differences in perceptual findings between our study and the perceptual results of other studies. All recovery protocols showing positive perceptual findings after CWI [20, 40, 41] did not implement full body immersion, with most using hip or umbilicus immersion in 10–12 °C water for 10 min compared to 14 min shoulder immersion (excluding the head and neck) at 15 °C in the current study.

In the current study ACT was unable to prevent a significant increase in soreness or a significant reduction in the perception of recovery at 1 and 24 h compared to baseline. At 1 h, the perception of recovery following ACT was also significantly worse than after CWT, as reported previously in a similar population of athletes [21]. During an ACT recovery participants are moving and expending energy so it is likely they do not yet feel recovered at 1 h post fatiguing exercise, the fitness level of the athletes may have also impacted upon their perceptual recovery after their use of ACT.

In the present study, in contrast to the perceptual results, average jump performance was hindered significantly at 1 h after CWI and COMB in comparison to CONT, ACT and respective baseline measures. It is likely that 1 h was not sufficient time for the muscles to rewarm, with a large number of participants noting stiffness in their legs when testing at 1 h post after the cold water strategies. Crowe and colleagues [43] reasoned that cold water may cause peripheral vasoconstriction and less blood flow to major muscle groups which combined with insufficient time for muscles to rewarm, could have attributed to the findings of the current study that indicated decreased power performance at 1 h and overall after water immersion recovery strategies. As in this study, Kinugasa and Kilding [18] found a combined recovery of ACT and CWI did not alter performance measures at 24 h in comparison to CONT and CWT recovery strategies for high performance youth soccer players. Other studies have shown CONT to be superior to CWI for cycling peak power and total work 1 h post-exercise [43] in a similar population of athletes as those in this study and 30 min post-exercise for swim performance in well-trained athletes [44].

In the current study despite not feeling recovered after ACT, the participants achieved the same jump power performance results as the CONT protocol which was significantly superior to CWI and COMB. The proposed mechanisms for enhanced recovery of performance after ACT in comparison to water immersion strategies include the enhanced rate of lactate removal via quicker lactate distribution to the liver and increased heart and skeletal muscle lactate utilization [45] and increased blood flow and accelerated recovery of interstitial creatine kinase levels [13].

The current study identified muscle soreness and TQR ratings to be significantly affected at 24 h (main time effects) in comparison to baseline and 48 h, but performance was not. Thus, it might be concluded from the findings of the present study that the fatiguing exercise was sufficient to induce perceptual decrements at 1 and 24 h but not performance decrements at 24 and 48 h. King and Duffield [21] also found similar findings with detrimental perceptual differences identified and performance unaffected at 24 h post fatiguing exercise in a similar population of athletes as those in this study.

A limitation of the present study is that a simulated team sport game and demands was used not an actual game. The fitness and ability of the participants of the current study is also a potential limitation, as it may not replicate that of high performance contact team sport athletes. As no significant differences were found at 24 and 48 h for performance measures, future research should examine recovery strategies at earlier time points after fatiguing exercise. Limb girths could also be investigated to examine the impact of recovery on swelling and osmotic fluid shifts. By examining swelling, the perceptual swelling (DALDA) differences that were found in this study could be investigated.

## Conclusion

The present study has identified that there are differences between recovery strategies for short term perceptual and performance recovery in non-elite athletes. For short term recovery, the present findings suggest that CWT elicited better perceptions of recovery, while the non-water based ACT and CONT strategies elicited better jump performance outcomes than CWI and COMB at 1 h post for non-elite athletes. Previously identified contributing mechanisms to explain these findings include influences of lactate clearance, stiffness, hydrostatic pressure and analgesic effects. It is recommended that future research further investigate these proposed recovery mechanisms for short term recovery from single and multiple bouts of fatiguing exercise in the hope of finding an optimal recovery strategy that can be confidently recommended for enhanced sporting performance.

## Abbreviations

ACT: Active recovery
COMB: A combined recovery of cold water immersion and active recovery
CONT: Control (passive) recovery
CWI: Cold water immersion
CWT: Contrast water therapy
DALDA: Daily Analysis of Life Demands of Athletes questionnaire
HR: Heart rate
RPE: Rating of perceived exertion
TQR: Total Quality Recovery scale
